# Development and Validation of the Quality-of-Life Assessment System for Lung Cancer Based on Traditional Chinese Medicine

**DOI:** 10.1155/2012/945910

**Published:** 2012-12-12

**Authors:** Chonghua Wan, Shengfu You, Peng Quan, Yi Song, Tao Liu, Jingen Lu, Peiyong Zheng

**Affiliations:** ^1^School of Humanities and Management and Research Center on Quality of Life and Applied Psychology, Guangdong Medical College, Dongguan 523808, China; ^2^Longhua Hospital, Shanghai University of Traditional Chinese Medicine, 725 South Wanping Road, Shanghai 200032, China

## Abstract

Traditional Chinese Medicine (TCM) has many unique features. Thequality-of-life (QoL) instrument for lung cancer based on Traditional Chinese Medicine (QLASTCM-Lu) was the first self-reported instrument specifically developed to assess the quality of life from the perspective of TCM. Structured group methods and theory in developmental rating scale were employed to establish a general and a specific module, respectively. Quantitative and qualitative data from 240 lung cancer patients were collected to assess the psychometric properties. The three identified scales of the QLASTCM-Lu (correspondence between man and universe, unity of the body and spirit, and lung cancer specific module) and the total score demonstrated excellent psychometric properties. Test-retest reliability of all domains ranged from 0.93 to 0.96, and internal consistency **α** ranged from 0.86 to 0.93. Correlation and factor analysis demonstrated good construct validity. Significant differences in the QLASTCM-Lu scales and total score were found among groups differing in TCM syndrome, supporting the clinical sensitivity of the QLASTCM-Lu. Statistically significant changes were found for each scale and the total score. Responsiveness of the QLASTCM-Lu was greater than that of QLQ-LC43. The QLASTCM-Lu is a psychometrically sound and clinically sensitive measure of quality of life for lung cancer patients, which can be applied to both TCM therapy and Western medicine therapy.

## 1. Introduction

Lung cancer has been the most common cancer in the world for several decades, and by 2008 there were an estimated 1.61 million new cases, representing 12.7% of all new cancers. The majority of the cases now occur in developing countries such as China (55%) [1]. With the development of new technology, the treatment of lung cancer has been greatly improved. However, the prognosis is not optimistic, and lung cancer continues to be the most common cause of cancer death [2]. How to improve quality of life has become the focus of lung cancer research. Clinical researchers are choosing measures of QoL as primary and secondary outcomes in clinical trials. Some quality-of-life questionnaires have been developed based on Western medical theories, such as EORTC QLQ-C43 (European Organization for Research and Treatment of Cancer Quality of Life Questionnaire, the QLQ-LC13 in conjunction with the QLQ-C30) [3–6], Functional Assessment of Cancer Therapy—Lung Cancer (FACT-L) [7], Lung Cancer Symptom Scale (LCSS) [8], and the Daily Diary Card (DDC) [9]. 

TCM has held an important position in health care in rural areas of China and is also valued in urban and well-developed areas because of its thousands years of heritage. There is an increasing need to measure QoL from the TCM perspective. Although TCM is based on a clear rationale and a well-established theoretical framework, it is also based on a different philosophical premise [10]. In Chinese medicine, the metaphoric views of the human body based on observations of nature are fully articulated in the theory of yin-yang [11]. The meaning of yin and yang in Chinese is bright and dark sides of an object. Chinese philosophy uses yin-yang to represent a wider range of opposite properties. In general, anything that is moving, ascending, bright, progressing, and hyperactive belongs to yang. The characteristics of stillness, descending, darkness, degeneration, hypoactivity belong to yin. Yin and yang are in conflict but at the same time mutually dependent. TCM emphasizes a balance and coordination of yin and yang. Within TCM philosophy, cancer results from a disturbance of yin-yang balance, and is a group of syndromes in which there is disharmony in the body-spirit-environment network [10]. The influences of environmental factors such as climatic condition and geographical locality have to be considered.

Although some scales have been developed based on Western medical theories, there is currently no validated, comprehensive, disease-specific measure to quantify QoL among lung cancer patients from a TCM perspective. A measurement tool to accurately and reliably quantify lung cancer patients' quality of life from a TCM perspective would be useful for both clinical and research purposes. To address this gap, we developed a novel, disease-specific health status instrument, the quality-of-life instrument for lung cancer based on Traditional Chinese Medicine (QLASTCM-Lu), to measure patients' perceptions of their symptoms, functional impairment, treatment concerns, and satisfaction with their health or clinical care, and to evaluate its psychometric properties in a large sample size.

## 2. Materials and Methods

The QLASTCM is composed of two basic elements: (1) a core questionnaire (QLASTCM-GM), comparing quality of life across various disease states and providing insight into improvements in general health, and (2) an additional disease-specific questionnaire (QLASTCM-Lu), designed to focus on domains, characteristics, and complaints most relevant to lung cancer.

### 2.1. Establishment of the General Module

**Figure 1:**
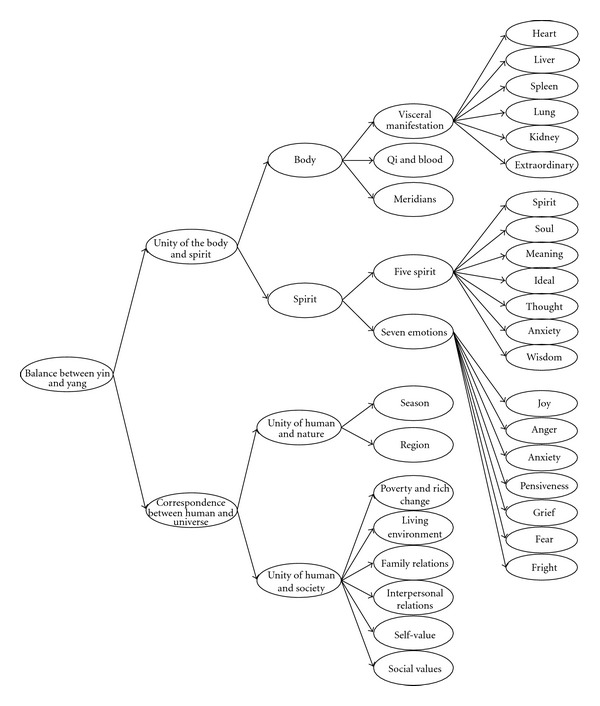
The theoretical construct of the QLASTCM-GM.

Based on a literature review of the QoL of lung cancer patients and TCM, the QLASTCM-GM should include, following two domains: “correspondence between man and universe” (*天人相应*, which means relevant adaptation of the human body to natural environment) and “unity of the body and spirit” (*形神一体*, which meansthe body is an organic whole). An overview of the theoretical construct of the QLASTCM-GM is shown in Figure 1.

The QLASTCM-GM was developed based on interviews with Chinese cancer patients and TCM professionals to generate 278 potentially candidate questions and create a conceptual framework for how clinical manifestations of lung cancer impact the lives of patients. Because there were no estimates of means or standard deviations for the new questionnaire, sample size was defined by the collective experience of the authors and no formal power calculations could be created. To establish the validity, reliability, and responsiveness of the QLASTCM-GM, 625 cancer patients were enrolled in a pilot test. To assess experts' perceptions of the importance of each potential item, the item pool was also administered to 50 clinical experts with a rating questionnaire that provided 5 responses, ranging from “not important” (1) to “extremely important” (5). An open-ended response item was also included, so experts could add issues that were not included on the original list. As shown in Table 1, the potential number of questions was 34 after distilling (T14, T25–27, T29–31, and T42 were deleted).

### 2.2. Establishment of the Specific Module

The development of the specific module was similar to the general module. 30 items about a few important categories such as physical symptoms, side effects, and psychology and social factors were proposed. After interviewing, importance analysis, and several rounds of expert group discussions, 13 items were selected and coded as F1 to F13. After that, 309 lung cancer patients were enrolled in the pilot test. The variation coefficient, correlation coefficient, cluster analysis, and the Cronbach coefficient method were used to screen each potential item. F10 was deleted.

To establish the validity, reliability, and responsiveness of the final questionnaire, we enrolled 240 lung cancer patients in the formal survey. After a brief introduction and explanation, questionnaires were distributed to patients and collected when completed. In order to get test-retest reliability, some randomly selected patients received a retest within the first 2-3 days. To observe the reaction degree and the change of quality of life, each patient received four longitudinal measures (pretreatment, 1 week, 3 months, and 6 months). As a collateral measure, the Chinese version of QLQ-LC43 [5] was also distributed to patients at the same time.

The scoring method in QLASTCM-Lu was similar to QLQ-LC43 consisting of five-point Likert responses ranging from the most severe symptoms to no symptoms. The positively stated items were directly scored from 1 to 5. The negatively stated items were scored in reverse. Scores in each domain were obtained by adding the within-domain item scores, and a total score was obtained by adding scale scores. 12 items inthe general module (T18, T19, T28, T32, T33, T34, T35, T36, T37, T38, T39, and T40) were directly scored, and the other 34 items were scored in reverse. The raw scores of 1 to 5 were transformed to a 0 to 100 scale, where a score of 0 indicated the most severe symptoms and a score of 100 indicated no limitation. Higher scores on the QLASTCM-Lu instrument indicated better quality-of-life status.

## 3. Results

### 3.1. Patient Characteristics

A total of 240 lung cancer patients (156 males) were enrolled, with a mean age of 60.3 ± 10.2 years (ranged from 32 to 87). 125 patients had junior middle school education, 70 had senior middle school education, 31 had community college education, and 14 had a four-year college education.

### 3.2. Content Validity

We conducted a series of analyses to generate an initial version of the instrument which was then used in the validation, reliability, and responsiveness testing reported in this paper. We began with a content specification and item generation phase, followed by a process of item reduction and refinement in which the instrument was reviewed by all participating personnel. The item pool was deemed to reflect the opinion of the World Health Organization regarding the connotation of quality of life [12]. The screening of items was also strictly programmed to achieve good content validity.

### 3.3. Construct Validity

Table 2 shows correlations between each item and its designated scale in bold type as well as correlations between each item and the other scales in normal type (unity of the body and spirit: T1–13, T15–24; correspondence between man and universe: T28, T32–41; lung cancer specific module: F1–9, F11–13). Correlations between each item and its designated scale were all significant at *P* < 0.001. The item-to-scale correlation of each item was high for the designated scale and weak for any other scale. T41, which showed a high correlation with “unity of the body and spirit” domain, is an exception. It hinted that T41 was included in the wrong domain, or its meaning would lead to misunderstanding. Finally, for all items, the item-to-total score correlation was higher than all item-to-scale correlations except the designated scale. This suggests a distinct separation of scales.

### 3.4. Convergent and Divergent Validity

We examined the convergent and divergent validity of QLASTCM-Lu by estimating its association with well-established questionnaires (QLQ-LC43). This was done by computing Pearson's correlation coefficients. We hypothesized that QLASTCM-Lu domains that assess “unity of the body and spirit” would correlate strongly with QLQ-LC43 physical function and emotional functioning domains, but poorly with the social function domain. As expected, results in Table 3 did show that QLASTCM-Lu “unity of the body and spirit” domain had higher correlations to QLQ-LC43 physical functioning (*r* = 0.81) and emotional functioning (*r* = 0.80) domains as compared with the social functioning (*r* = 0.63) domain. Table 3 further confirms the direction of the correlation as expected. These results indicated that the convergent and divergent validity was high. Conversely, QLASTCM-Lu “correspondence between man and universe” domain had lower correlations to all QLQ-LC43 domains. This provided evidence that “correspondence between man and universe” domain was a unique domain which reflected traditional Chinese culture.

### 3.5. Clinical Validity

It is well known that the clinical feature would affect quality of life. To establish QLASTCM-Lu's sensitivity to TCM syndromes, as per clinicians' assessments, we compared the mean scores according to professionals' clinical categorization of patients into six basic TCM syndromes (“syndrome of phlegm dampness due to spleen deficiency,” “yin deficiency syndrome toxic heat,” “syndrome of deficiency of both qi and yin,” “type of qi-stagnancy and blood stasis,” “deficiency of lung-spleen qi,” and others). As seen in Table 4, there was no statistical difference before treatment and a statistically difference after 3-month treatment. This indicates that the clinical validity of QLASTCM-Lu was good enough to reflect the differences between different TCM syndromes.

### 3.6. Reliability

Internal consistency was examined using reliability coefficients (Cronbach *α* coefficients), which were calculated from the data from the first measure. The intraclass correlation coefficient (ICC) was used to assess test-retest reliability, which was calculated from 48 patients' data collected from the second test in the next day (Table 5).

### 3.7. Responsiveness

Standardized Response Means (SRM) were used to assess clinically meaningful changes (Table 6). 240 patients were randomly selected for a retest to evaluate the responsiveness after three months of treatment. SRM was calculated by using the paired *t*-test. The difference between baseline and three months was statistically significant in most domains (all except general module). However, the SRM of the QLQ-LC43 was not statistically significant in all domains, which means that the QLASTCM-Lu was more sensitive than the QLQ-LC43. There was no statistical difference in the QLASTCM-Lu general module which might be attributed to the reversal of “unity of the body and spirit” and “correspondence between man and universe.”

## 4. Discussion

This paper describes the development and validation of QLASTCM-Lu which consists of 46 items and three scales: unity of the body and spirit (23 items), correspondence between man and universe (11 items), and lung cancer specific module (12 items). The QLASTCM-Lu was created partly in response to feedback from TCM professionals who felt that existing measures were not suitable for Chinese patients using TCM cancer therapy. Quality of lifeisasubjective concept which was often evaluated through personal feelings or one's own evaluation. It is well known that culture contributes to quality of life. Any-quality-of life measures will only apply to a defined community. Chinese culture is different from Western culture. The basis of TCM diagnosis includes palpitation, upset, choler, lethargy, night sweating, and xerostomia, which are not included in Western medicine. The focus of TCM is on the patient rather than the disease and fundamentally aims to promote health while enhancing quality of life with therapeutic strategies for treatment of specific diseases in a holistic fashion [13].

The validity of the QLASTCM-Lu was evaluated by content validity, construct validity, and criterion-related validity. The usual methods to evaluate content validity were correlation coefficient, factor analysis, and Structural Equation Model (SEM) [14]. As the number of items was not sufficient, SEM was not used in this study [15]. Content validity was evaluated using the Delphi method. Based on results from correlation analysis and exploratory factor analysis, the construct validity was good. When no clear gold standard exists for quantifying a property, the most assuring method to establish the validity of the QLASTCM-Lu is convergent validity in which the new measure is shown to be correlated with other measures that are believed to quantify the same concept. Such correlations are considered to be high when the correlation coefficient is ≥0.4. Conversely, divergent validity is demonstrated when domain items that are thought to measure different concepts have low correlations (*r* < 0.4). Correlation between the same or similar domain of two questionnaires was higher than that from the different domains. The convergent validity and divergent validity were satisfied in this study.

Known group validity assesses whether the QLASTCM-Lu discriminates between clinically different groups. QLASTCM-Lu total scores were evaluated and compared between patients grouped according to physicians' clinical evaluation. Physicians' clinical assessment of their patients' syndrome was explicitly collected on the case report forms. Patients with a particular TCM syndrome were compared. The results showed good clinical validity of the QLASTCM-Lu.

Internal consistency, or reliability, examines the consistency of items within a scale and quantifies the degree to which each item is measuring aspects of the same underlying domain [16]. In this analysis the internal consistency of the QLASTCM-Lu and its subscales was examined using Cronbach *α* coefficient in which a value of 0.90 or higher is excellent and 0.80 or higher is sufficient [17]. The test-retest reliability was evaluated by ICC, which measures how stable responses are over time, which was calculated from 48 patients' data from the second test during the following day, in which patients' quality-of-life status would be less likely to change. The internal consistency reliability and test-retest reliability of the QLASTCM-Lu are both higher than 0.8. It was concluded that the QLASTCM-Lu possesses good reliability and stability.

The responsiveness of an instrument refers to its ability to detect clinically meaningful changes in a patient's quality-of-life status over time. We selected SRM as a measure of change in instruments' scores within each group and calculated it for all questionnaires. SRM is defined as mean score change divided by the standard deviation of that score change. As a benchmark for the interpretation of SRM, Cohen describes an effect size of 0.20 as small, 0.50 as medium, and 0.80 as large [18]. A series of paired *t*-tests were conducted to compare changes in scores for all questionnaires. The QLASTCM-Lu was considered to have good responsiveness.

The QLASTCM-Lu shares some common characteristics with the QLQ-LC43. For example, specific module and general modules were developed at the same time. Items in both measures were rated using a Likert scoring system. However, there were some distinguishing characteristics in the QLASTCM-Lu. From the view of the structure, the QLQ-LC43 general module (QLQ-C30) was constituted by nine scales and six single items [4]. There are a large number of single items on the reaction symptoms in the QLQ-C30. The QLASTCM-Lu general module was constituted by 34 items from only 2 scales. Moreover, there are only 13 items in the QLQ-LC43 specific module (QLQ-LC13), and it is analyzed in 10 dimensions. Only one dimension needs to be analyzed in the QLASTCM-Lu specific module. In other words, scoring for the QLASTCM-Lu is easier. Specifically, items in the QLASTCM-Lu capture significant Chinese cultural characteristics, such as “I feel sore and weak in my waist and knees” (T7), “I am happy with the surrounding natural environment” (T28), “I am happy with the climate of residence” (T32), and “I am afraid of wind” (F7). These items embody “correspondence between man and universe,” which is specially focused in Chinese culture, and are not included in other questionnaires.

Although this study successfully developed a new quality-of-life measure and subsequently validated this instrument, there are some limitations in this study. One limitation is that the responsiveness of the QLASTCM-Lu is not high enough. It might be that the scale was too large (there were 23 items in “unity of the body and spirit”) and that this counteractssome minor facets under the main domain. Minor components should be subdivided under main domains. Another limitation is that our survey excluded very ill patients; therefore, the results may not be applicable to these special groups. There were also a relatively higher proportion of some TCM syndromes in our sample, for instance, 2 out of 6 syndromes (i.e., “syndrome of phlegm dampness due to spleen deficiency” and “syndrome of deficiency of both qi and yin”) were noted in 68% patients, while another 4 syndromes were only noted in 32%of the patients. Fortunately, the bias is expected to be consistent for all subjects and should not affect our conclusions.

Future research is being planned concerning the interpretation and application of the QLASTCM-Lu in different samples. Our goals are to establish separate norms for different TCM syndromes in order to determine clinically meaningful changes in QLASTCM-Lu scores.

## 5. Conclusions

On the basis of the above findings, the three identified scales of the QLASTCM-Lu and the total QLASTCM-Lu score demonstrated excellent psychometric properties. We recommend the use of the QLASTCM-Lu for the following reasons. The development of the QLASTCM-Lu has significant Chinese cultural characteristics. The concrete items in the QLASTCM-Lu are popular and straightforward, and it can be applied to both TCM therapy and Western medicine therapy. In our opinion, the QLASTCM-Lu would make a useful addition to the assessment protocol of clinical trials for lung cancer.

## Figures and Tables

**Table 1:** Reduction of the QLASTCM-Lu.

Items	English	Chinese	Remain or not
T1	I feel lack of strength constantly	*我经* *常有* *力不* *从心* *的感* *觉*	*√*
T2	It gets difficult as I walk short distance on foot	*短距* *离步* *行时*, *我感到困难*	*√*
T3	It gets difficult as I walk long distance on foot	*长距* *离步* *行时*, *我感到困难*	*√*
T4	I lie down or sit down due to lack of energy in the daytime	*我白* *天因* *精力* *差需* *要卧* *床或* *坐在* *椅子* *上*	*√*
T5	Hard work will add to my discomfort	*劳累* *会加* *重我* *的不* *适*	*√*
T6	I feel dizzy	*我感* *觉头* *晕*	*√*
T7	I feel sore and weak in my waist and knees	*我感* *觉腰* *膝酸* *软*	*√*
T8	I lose my appetite	*我感* *觉没* *有胃* *口*	*√*
T9	I feel sick	*我感* *觉恶* *心*	*√*
T10	I vomit	*我有* *呕吐*	*√*
T11	I have constipation	*我有* *便秘*	*√*
T12	I feel that I am losing weight	*我感* *觉自* *己越* *来越* *消瘦*	*√*
T13	I am more likely to have other diseases	*我更* *容易* *得其* *他病*	*√*
T14	My limbs are agile	*我的* *肢体* *活动* *自如*	×
T15	I can not sleep	*我有* *失眠*	*√*
T16	I feel that I do not get enough sleep	*我觉* *得睡* *不够*	*√*
T17	I am forgetful	*我* *容易* *健* *忘*	*√*
T18	My mind is active	*我觉* *得自* *己思* *维活* *跃*	*√*
T19	I am in a good mood	*我觉* *得心* *情愉* *快*	*√*
T20	I am irritated	*我变* *得急* *躁*	*√*
T21	I am overconcerned about my disease and could not get over with it	*我过* *度关* *心自* *己的* *疾病* *并且* *不能* *自控*	*√*
T22	I feel sad	*我感* *觉悲* *伤*	*√*
T23	I often sigh	*我会* *唉声* *叹气*	*√*
T24	I am worried about my sickness	*我担* *心自* *己的* *病情* *会变* *糟*	*√*
T25	I do not feel comfortable with chemotherapy	*化疗* *让* *我感* *觉* *不舒* *服*	×
T26	I am satisfied with TCM therapies	*我对* *中医* *药治* *疗满* *意*	×
T27	I live in a safe and harmonious place	*我觉* *得自* *己居* *住在* *一个* *安全* *和谐* *的环* *境里*	×
T28	I am happy with the surrounding natural environment	*我对* *周围* *的自* *然环* *境满* *意*	*√*
T29	I can adapt to climate change of residence	*我能* *够适* *应居* *住地* *气候* *的变* *化*	×
T30	I am comfortable with the place I stay	*我觉* *得住* *的地* *方舒* *适*	×
T31	I am fond of the place I stay	*我喜* *欢自* *己住* *的地* *方*	×
T32	I am happy with the climate of residence	*我对* *自* *己居* *住地* *的* *气候* *满意*	*√*
T33	I am confident in the therapy	*我对* *接受* *的治* *疗充* *满信* *心*	*√*
T34	I am satisfied with my performance	*我对* *自* *己的* *工* *作表* *现感* *到* *满意*	*√*
T35	I am satisfied with my ability to support my family	*我对* *自己* *供养* *或支* *持家* *人的* *能力* *感到* *满意*	*√*
T36	I feel good about my health	*我对* *自* *己的* *身* *体状* *况* *满意*	*√*
T37	I feel happy	*我觉* *得自* *己幸* *福*	*√*
T38	I am emotionally supported by my family/friends/coworkers	*我在* *感情* *上能* *得到* *家人* (*朋友同事*) *的关怀*	*√*
T39	I have friends who I can confide with	*我有* *谈心* *的朋* *友*	*√*
T40	I am content with my interpersonal relationships	*我对* *自* *己的* *人* *际关* *系* *满意*	*√*
T41	My health status or therapy hinders my social activity	*我的* *身* *体状* *况* *或治* *疗过* *程*, *妨碍了我的社交活动*	*√*
T42	My health status or therapy results to my financial problem	*我的* *身* *体状* *况* *或治* *疗过* *程*, *造成了我的经济困难*	×
F1	I feel dyspnea	*我感* *到呼* *吸困* *难*	*√*
F2	I feel short of breath	*我感* *到气* *短*	*√*
F3	I am experiencingchest distress	*我感* *到胸* *闷*	*√*
F4	I feel chest pain	*我感* *到胸* *痛*	*√*
F5	I cough	*我有* *咳嗽*	*√*
F6	I tend to sweat after usual activities	*我平* *时稍* *微* *活动* *就* *容易* *出汗*	*√*
F7	I am afraid of wind	*我怕* *风*	*√*
F8	I am thirsty	*我感* *觉口* *干*	*√*
F9	I sweat during sleep	*我睡* *着时* *出汗*, *醒来后停止*	*√*
F10	I feel hot in my cheeks in afternoons or evenings	*我下* *午或* *晚间* *感觉* *面部* *发红*	×
F11	I feel phlegm in the throat	*我感* *觉咽* *喉有* *痰*	*√*
F12	My voice sounds hoarse	*我声* *音嘶* *哑*	*√*
F13	I cough blood	*我有* *咳血*	*√*

*Deleted items after distillation ×.

**Remain items after distillation *√*.

**Table 2:** Correlation coefficients among items and domains of QLASTCM-Lu (*n* = 240).

Items	Unity of the body and spirit	Correspondence between man and universe	Lung cancer-specific module	General module	Total score
T1	**0.74**	0.26	0.63	0.64	0.67
T2	**0.81**	0.39	0.72	0.75	0.78
T3	**0.72**	0.40	0.65	0.68	0.71
T4	**0.72**	0.24	0.62	0.62	0.65
T5	**0.71**	0.24	0.66	0.62	0.66
T6	**0.54**	0.19	0.46	0.47	0.49
T7	**0.54**	0.10	0.48	0.43	0.47
T8	**0.55**	0.22	0.44	0.49	0.50
T9	**0.58**	0.27	0.50	0.53	0.55
T10	**0.57**	0.33	0.44	0.55	0.55
T11	**0.56**	0.28	0.52	0.52	0.55
T12	**0.65**	0.33	0.55	0.61	0.62
T13	**0.69**	0.32	0.52	0.63	0.63
T15	**0.68**	0.19	0.53	0.57	0.59
T16	**0.39**	0.08	0.30	0.31	0.32
T17	**0.67**	0.23	0.61	0.58	0.62
T18	**0.32**	0.57	0.20	0.47	0.42
T19	**0.50**	0.63	0.30	0.62	0.56
T20	**0.68**	0.39	0.53	0.65	0.65
T21	**0.64**	0.37	0.52	0.61	0.62
T22	**0.75**	0.45	0.59	0.73	0.73
T23	**0.74**	0.42	0.61	0.71	0.71
T24	**0.73**	0.44	0.60	0.71	0.72
T28	0.30	**0.65**	0.25	0.48	0.44
T32	0.04	**0.41**	0.03	0.20	0.15
T33	0.37	**0.79**	0.24	0.59	0.52
T34	0.43	**0.67**	0.39	0.59	0.56
T35	0.44	**0.75**	0.40	0.62	0.59
T36	0.54	**0.73**	0.46	0.69	0.66
T37	0.43	**0.77**	0.31	0.63	0.57
T38	0.08	**0.64**	0.05	0.32	0.24
T39	0.09	**0.56**	0.01	0.30	0.24
T40	0.23	**0.70**	0.11	0.46	0.39
T41	0.61	**0.34**	0.57	0.58	0.61
F1	0.66	0.32	**0.74**	0.61	0.68
F2	0.65	0.34	**0.73**	0.61	0.67
F3	0.63	0.36	**0.73**	0.60	0.67
F4	0.50	0.36	**0.66**	0.51	0.58
F5	0.45	0.26	**0.59**	0.43	0.50
F6	0.54	0.18	**0.64**	0.46	0.53
F7	0.61	0.28	**0.70**	0.55	0.62
F8	0.47	0.09	**0.58**	0.38	0.45
F9	0.43	0.17	**0.55**	0.38	0.44
F11	0.51	0.24	**0.65**	0.46	0.53
F12	0.35	0.09	**0.46**	0.29	0.35
F13	0.49	0.28	**0.55**	0.47	0.51

*Correlations between each item and its designated scale are in bold type.

**Table 3:** Correlation coefficients between items of QLASTCM-Lu and QLQ-LC43 (*n* = 240).

QLQ-LC43	QLASTCM-Lu				
	Unity of the body and spirit	Correspondence between man and universe	Specific module	General module	Total score
Physical functioning	0.81	0.43	0.76	0.73	0.79
Role functioning	0.68	0.40	0.66	0.67	0.69
Emotional functioning	0.80	0.51	0.79	0.67	0.79
Cognitive functioning	0.75	0.36	0.69	0.64	0.71
Social functioning	0.63	0.45	0.64	0.60	0.66
Global health status/QoL	0.71	0.51	0.73	0.65	0.74
Fatigue	−0.78	−0.33	−0.70	−0.69	−0.73
Nausea and vomiting	−0.61	−0.32	−0.57	−0.57	−0.60
Pain	−0.68	−0.42	−0.67	−0.61	−0.69
Dyspnoea	−0.78	−0.40	−0.73	−0.76	−0.77

**Table 4:** QoL of different TCM syndromes after three-month treatment.

Traditional Chinese Medicine syndrome	*n *	Unity of the body and spirit	Correspondence between man and universe	Specific module	General module	Total score
Syndrome of phlegm dampness due to spleen deficiency	85	69.80 ± 10.41	60.90 ± 11.70	69.22 ± 15.53	66.92 ± 5.86	67.52 ± 7.68
Yin deficiency syndrome toxic heat	19	69.64 ± 10.32	59.58 ± 10.63	67.11 ± 12.74	66.39 ± 7.68	66.58 ± 8.35
Syndrome of deficiency of both qi and yin	79	68.51 ± 10.73	62.83 ± 9.39	68.26 ± 15.46	66.67 ± 7.16	67.08 ± 8.69
Type of qi-stagnancy and blood stasis	34	75.33 ± 10.31	57.09 ± 10.11	76.96 ± 14.58	69.43 ± 5.73	71.39 ± 7.58
Deficiency of lung-spleen qi	9	58.83 ± 0.38	68.75 ± 4.82	53.13 ± 2.95	62.04 ± 1.30	59.71 ± 0.19
Others	14	70.81 ± 10.55	58.93 ± 14.14	72.02 ± 15.17	66.96 ± 9.03	68.28 ± 9.44

		*F* = 3.95 *P* = 0.002	*F* = 2.33 *P* = 0.043	*F* = 3.83 *P* = 0.002	*F* = 1.88 *P* = 0.099	*F* = 3.12 *P* = 0.010

*Mean ± SD.

**Table 5:** Reliability of QLASTCM-Lu.

Domain/facet	Correlation coefficient *r *	ICC	Cronbach *α* coefficient
	(*n* = 48)	(*n* = 48)	(*n* = 240)
Unity of the body and spirit	0.96	0.96 (0.94–0.98)	0.93
Correspondence between man and universe	0.93	0.93 (0.88–0.96)	0.85
Specific module	0.95	0.95 (0.91–0.97)	0.86
General module	0.96	0.96 (0.93–0.98)	0.93

Total score	0.96	0.96 (0.93–0.98)	—

**Table 6:** Responsiveness of QLASTCM-Lu and QLQ-LC43 (*n* = 240).

Domain/facet	Baseline	Three months	*t *	*P *	SRM
QLASTCM-Lu					
Unity of the body and spirit	76.01 ± 15.04	69.84 ± 10.68	6.11	0.000	0.41
Correspondence between man and universe	52.18 ± 17.99	61.07 ± 10.82	−6.49	0.000	0.43
Specific module	79.81 ± 13.79	69.50 ± 15.41	8.88	0.000	0.59
General module	68.31 ± 14.17	67.00 ± 6.64	1.53	0.129	0.10
Total	71.31 ± 13.43	67.65 ± 8.25	4.36	0.000	0.29

QLQ-C30					
Physical functioning	77.65 ± 18.97	78.96 ± 8.25	−1.07	0.285	0.07
Role functioning	76.12 ± 26.90	73.21 ± 13.55	1.61	0.109	0.11
Emotional functioning	79.84 ± 21.56	76.41 ± 11.57	2.42	0.017	0.16
Cognitive functioning	83.63 ± 20.88	82.29 ± 11.55	0.92	0.359	0.06
Social functioning	68.45 ± 25.78	69.72 ± 14.62	−0.75	0.455	0.05
Global health status/QoL	59.38 ± 24.46	59.78 ± 13.58	−0.24	0.808	0.02
Fatigue	30.46 ± 22.26	29.91 ± 9.75	0.36	0.720	0.02
Nausea and vomiting	7.74 ± 15.04	5.21 ± 9.86	2.08	0.038	0.14
Pain	23.44 ± 24.50	24.48 ± 15.21	−0.62	0.533	0.04
Dyspnoea	22.77 ± 24.50	27.38 ± 16.23	−2.52	0.012	0.17
Insomnia	25.15 ± 34.97	25.45 ± 16.47	−0.13	0.897	0.01
Appetite loss	16.22 ± 22.32	23.21 ± 17.77	−4.13	0.000	0.28
Constipation	12.20 ± 18.94	6.25 ± 13.42	3.97	0.000	0.27
Diarrhoea	8.18 ± 31.21	2.53 ± 8.85	2.61	0.010	0.17
Financial difficulties	28.27 ± 28.48	31.99 ± 19.02	−1.83	0.069	0.12

QLQ-LC13					
Dyspnoea	20.88 ± 20.04	19.15 ± 10.12	1.31	0.191	0.09
Coughing	29.17 ± 26.83	29.91 ± 16.19	−0.38	0.703	0.03
Haemoptysis	9.08 ± 18.19	3.27 ± 10.43	4.52	0.000	0.30
Sore mouth	4.32 ± 13.98	0.74 ± 4.94	3.55	0.000	0.24
Dysphagia	6.85 ± 17.08	2.98 ± 9.53	2.96	0.003	0.20
Peripheral neuropathy	7.89 ± 18.20	3.72 ± 10.98	2.94	0.004	0.20
Alopecia	13.54 ± 23.41	10.27 ± 20.67	1.65	0.101	0.11
Pain in chest	21.88 ± 24.91	23.81 ± 17.54	−1.10	0.275	0.07
Pain in arm/shoulder	12.35 ± 20.49	5.80 ± 13.43	3.95	0.000	0.26
Pain in other parts	8.18 ± 19.14	2.38 ± 9.69	4.40	0.000	0.29

*Mean ± SD.

## References

[1] Ferlay J, Shin HR, Bray F, Forman D, Mathers C, Parkin DM (2010). Estimates of worldwide burden of cancer in 2008: GLOBOCAN 2008. *International Journal of Cancer*.

[2] Siegel R, Ward E, Brawley O, Jemal A (2011). Cancer statistics, 2011: the impact of eliminating socioeconomic and racial disparities on premature cancer deaths. *CA Cancer Journal for Clinicians*.

[3] Fayers P, Bottomley A (2002). Quality of life research within the EORTC—the EORTC QLQ-C30. *European Journal of Cancer*.

[4] Fayers PM, Weeden S, Curran D (2001). *EORTC QLQ-C30 Scoring Manual*.

[5] Wan C, Zhang C, Tu X (2008). Validation of the simplified Chinese version of the quality of life instrument EORTC QLQ-LC43 for patients with lung cancer. *Cancer Investigation*.

[6] Chie WC, Yang CH, Hsu C, Yang PC (2004). Quality of life of lung cancer patients: validation of the Taiwan Chinese version of the EORTC QLQ-C30 and QLQ-LC13. *Quality of Life Research*.

[7] Cella D, Eton DT, Fairclough DL (2002). What is a clinically meaningful change on the Functional Assessment of Cancer Therapy-Lung (FACT-L) questionnaire?: results from Eastern Cooperative Oncology Group (ECOG) study 5592. *Journal of Clinical Epidemiology*.

[8] Gralla RJ, Edelman MJ, Detterbeck FC (2009). Assessing quality of life following neoadjuvant therapy for early stage non-small cell lung cancer (NSCLC): results from a prospective analysis using the Lung Cancer Symptom Scale (LCSS). *Supportive Care in Cancer*.

[9] Fayers PM, Bleehen NM, Girling DJ, Stephens RJ (1991). Assessment of quality of life in small-cell lung cancer using a Daily Diary Card developed by the Medical Research Council lung cancer working party. *British Journal of Cancer*.

[10] Xu W, Towers AD, Li P, Collet JP (2006). Traditional Chinese medicine in cancer care: perspectives and experiences of patients and professionals in China. *European Journal of Cancer Care*.

[11] Tcmpage.com http://www.tcmpage.com/.

[12] Kuyken W (1995). The World Health Organization Quality of Life Assessment (WHOQOL): position paper from the World Health Organization. *Social Science and Medicine*.

[13] Patwardhan B, Warude D, Pushpangadan P, Bhatt N (2005). Ayurveda and traditional Chinese medicine: a comparative overview. *Evidence-Based Complementary and Alternative Medicine*.

[14] Haynes SN, Richard DCS, Kubany ES (1995). Content validity in psychological assessment: a functional approach to concepts and methods. *Psychological Assessment*.

[15] Ullman JB, Bentler PM (2003). Structural equation modeling. *Handbook of Psychology*.

[16] Henson RK (2001). Understanding internal consistency reliability estimates: a conceptual primer on coefficient alpha. *Measurement and Evaluation in Counseling and Development*.

[17] Streiner DL, Norman GR (1995). *Health Measurement Scales: A Practical Guide to Their Development and Use*.

[18] Husted JA, Cook RJ, Farewell VT, Gladman DD (2000). Methods for assessing responsiveness: a critical review and recommendations. *Journal of Clinical Epidemiology*.

